# Supplementary material for: Anti-microRNA screen uncovers miR-17 family within miR-17~92 cluster as the primary driver of kidney cyst growth
Source: Sci Rep. 2019 Feb 13;9:1920. doi: 10.1038/s41598-019-38566-y (PMC6374450; doi:10.1038/s41598-019-38566-y)
Supplement: Supplementary file 1 — Supplementary Info [file 41598_2019_38566_MOESM1_ESM.docx]

Manuscript Title:

**Anti-microRNA screen uncovers miR-17 family within miR-17~92 cluster as the primary driver of kidney cyst growth**

Matanel Yheskel^1^, Ronak Lakhia^1^, Patricia Cobo-Stark, Andrea Flaten^1^, and Vishal Patel^1^

^1^Department of Internal Medicine, Division of Nephrology, University of Texas Southwestern Medical Center, Dallas, Texas

Address correspondence to:

Vishal Patel, M.D.

Department of Internal Medicine/Nephrology

University of Texas Southwestern Medical Center

5323 Harry Hines Blvd.

Dallas, Texas, USA 75390

Email: [vishald.patel@utsouthwestern.edu](mailto:vishald.patel@utsouthwestern.edu)

Running Title: Anti-miR-17 treatment slows cyst growth in ADPKD.

**Table of Contents:**

1. **Supplementary Figure 1**
2. **Supplementary Figure 2**
3. **Supplementary Figure 3**
4. **Supplementary Figure 4**
5. **Supplementary Table 1**
6. **Supplementary Table 2**
7. **Supplementary Table 3**


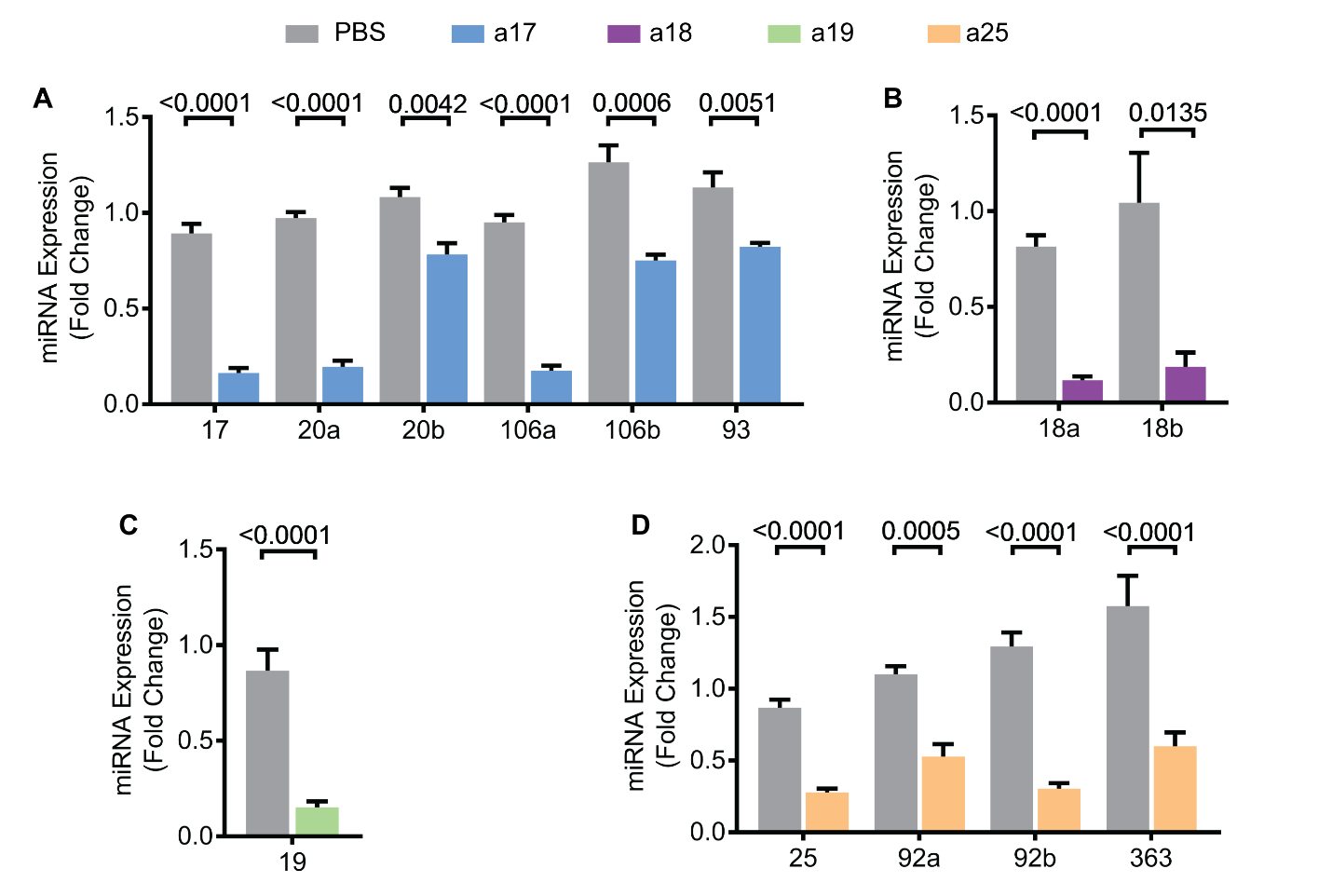


**Supplementary Figure 1: Anti-miRs significantly reduced expression of miRNAs in each target family.** Q-PCR analysis was performed to determine the expression of each miRNA in the **(A)** miR-17, **(B)** miR-18, **(C)** miR-19, and **(D)** miR-25 families after inhibition with their respective anti-miR cocktails. Data are presented as mean ± SEM. N=5 per group. Statistical analyses: Student’s t-test, ns indicates *P* >0.05.

**Supplementary Information**


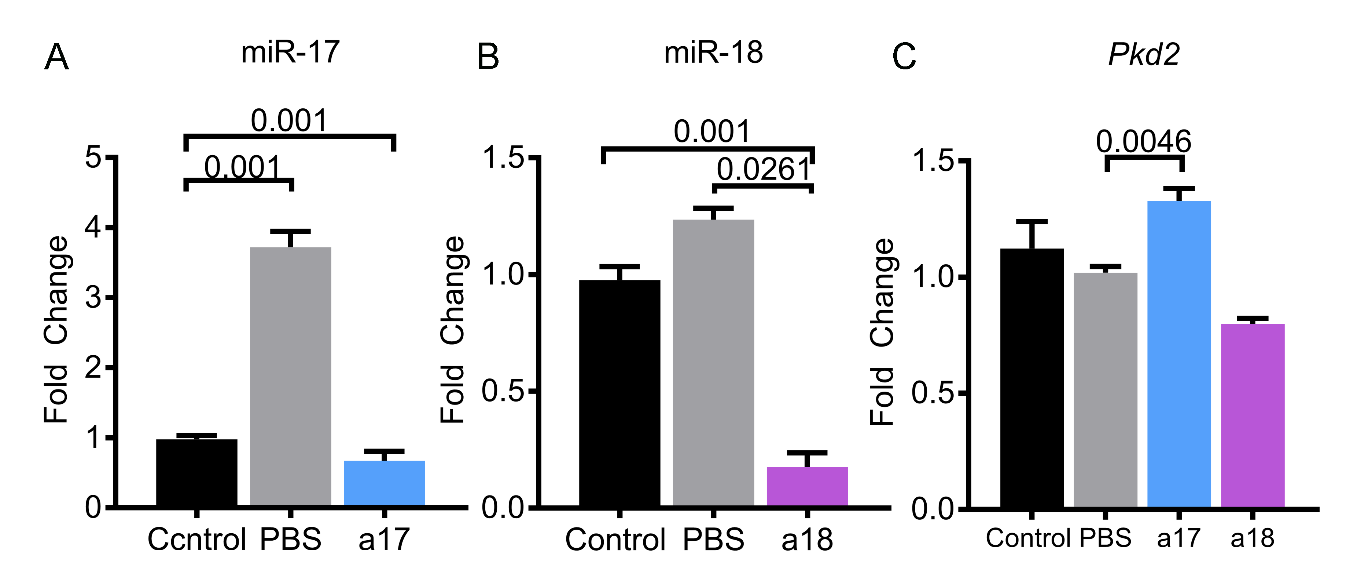


**Supplementary Figure 2:** Q-PCR analysis was performed to determine the expression of miR-17 (A) and miR-18 (B) in kidneys from wild-type (black), PBS-treated *Pkd1*-KO (grey), anti-miR-17-treated *Pkd1*-KO (blue) or anti-miR-18-treated *Pkd1*-KO mice (purple). Anti-miRs reduced expression of target miRNAs by 50-80% of wild-type level. Q-PCR analysis was performed to determine the expression of *Pkd2* (C), a direct miR-17 target, in kidneys from wild-type (black), PBS-treated *Pkd1*-KO (grey), anti-miR-17-treated *Pkd1*-KO (blue) or anti-miR-18-treated *Pkd1*-KO mice (purple). *Pkd2* expression was increased after anti-miR-17 treatment indicating functional inhibition of miR-17. Data are presented as mean ± SEM. N=5 per group. Statistical analyses: Student’s t-test, ns indicates *P* >0.05.

**
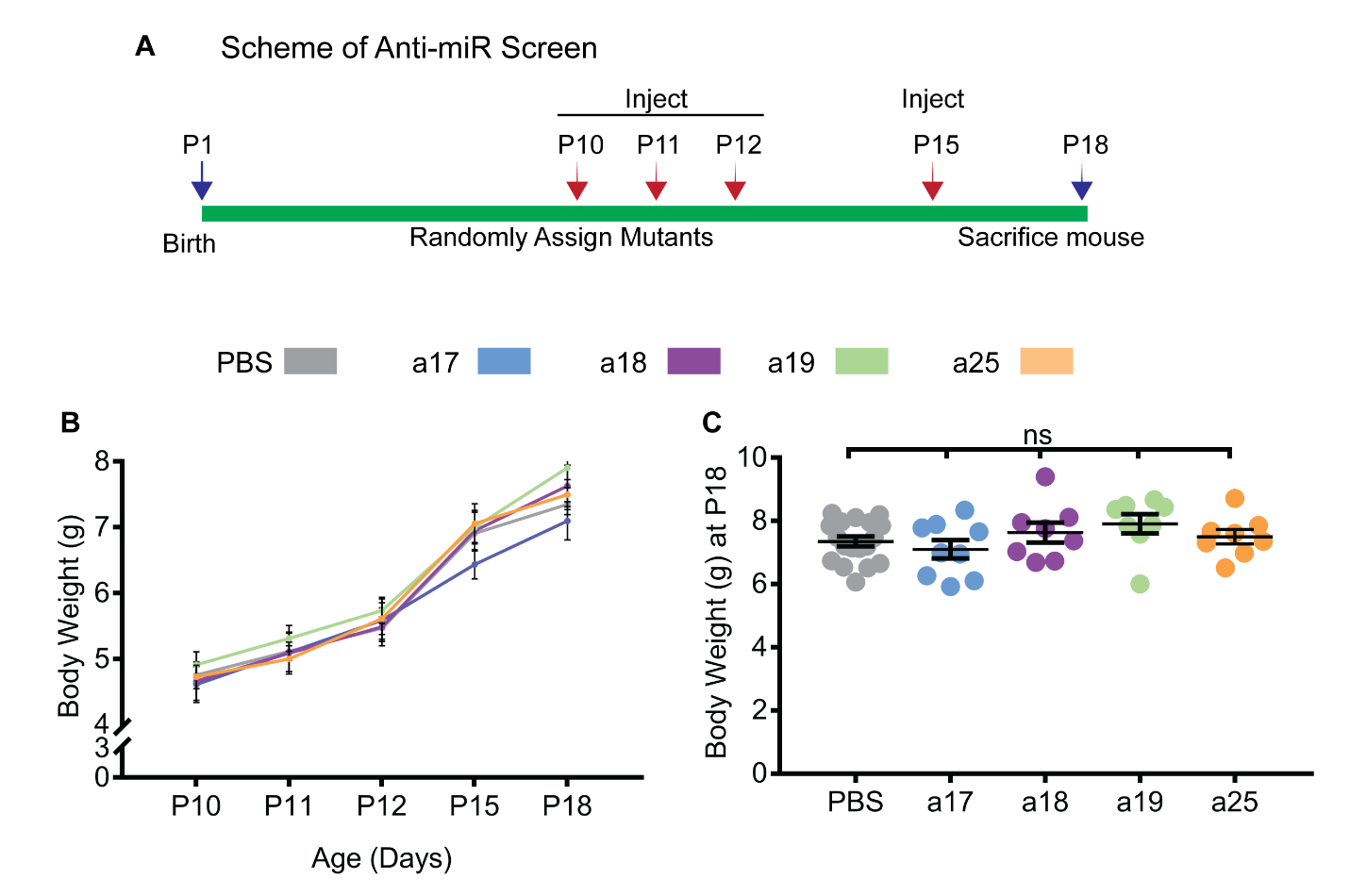
Supplementary Figure 3: (A)** Scheme of anti-miR screen in *Pkd1*-KO mice. Mice were randomly assigned to receive either PBS, anti-miR-17 (a17), anti-miR-18 (a18), anti-miR-19 (a19), or anti-miR-25 (a25). The total anti-miR dose per injection was 20 mg/kg. Each mouse was injected at P10, P11, P12, and P15, and sacrificed at P18. **(B)** Body weights were monitored during anti-miR treatment. There were no significant changes in body weights between treatment arms throughout the study. **(C)** Body weight of each mouse on P18 prior to sacrifice. There was no difference in body weight between groups. Statistican analysis: One-way ANOVA (post hoc analysis: Dunnett’s multiple comparisons test). ns indicates *P*>0.05.


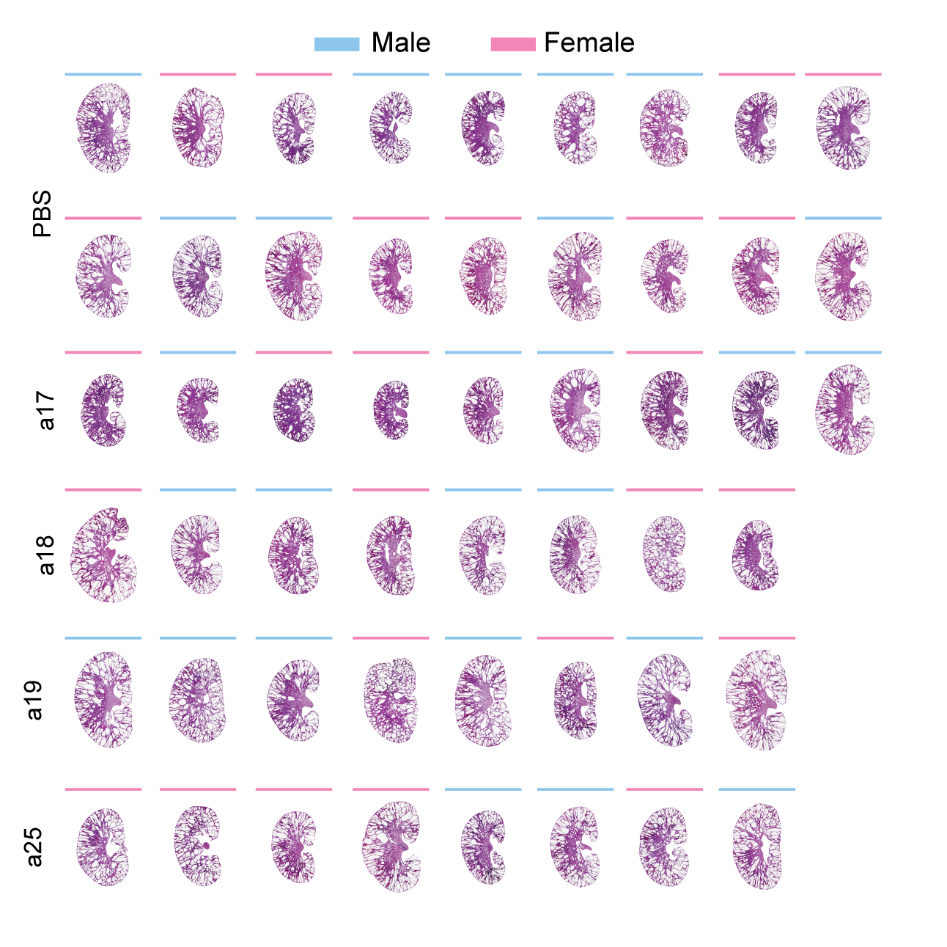
**Supplementary Figure 4:** H&E kidney sections for all mice sacrificed in this study. PBS n=18, anti-miR-17 (a17) n=9, anti-miR-18 (a18) n=8, anti-miR-19 (a19) n=8, and anti-miR-25 (a25) n=8.


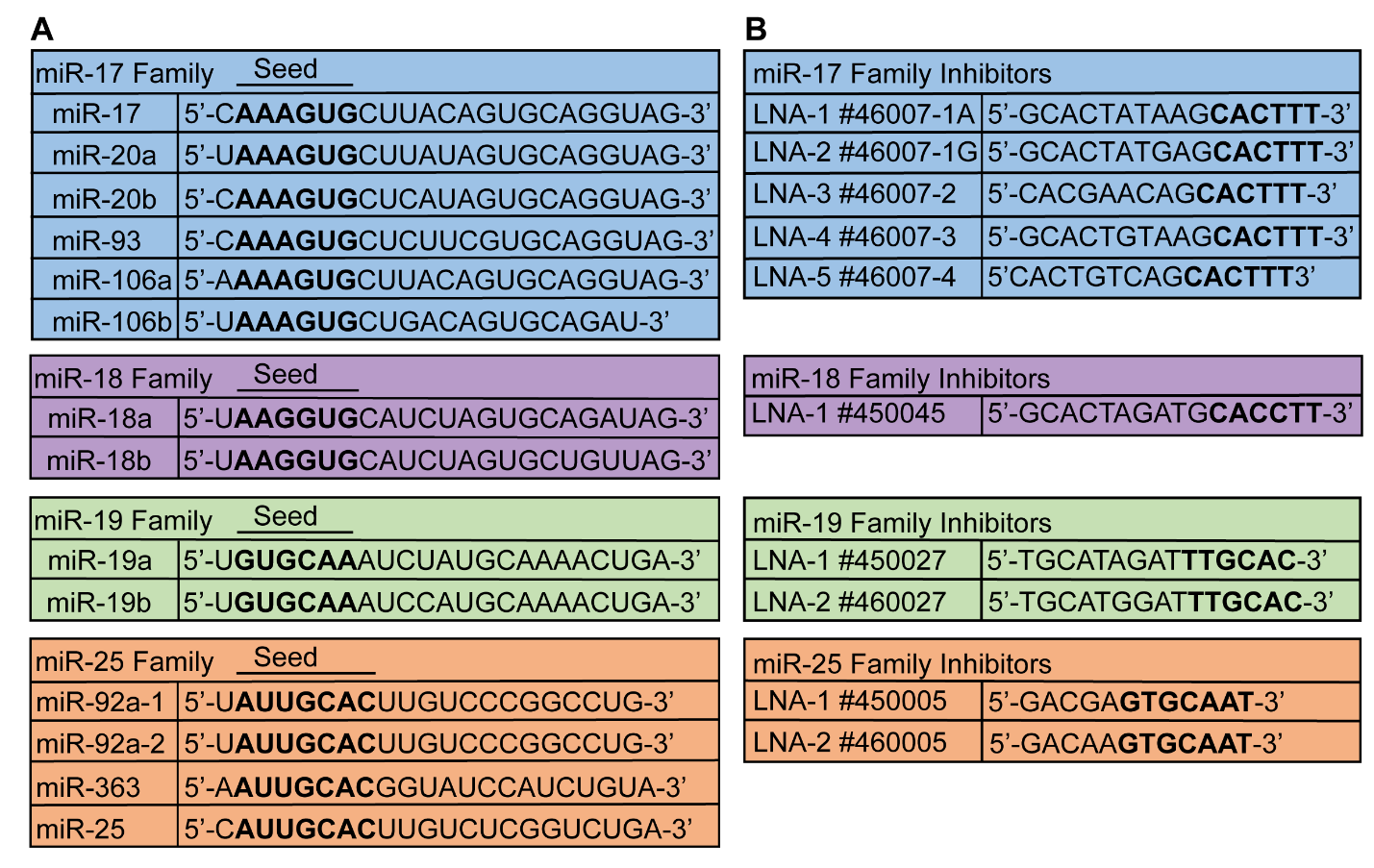


**Supplementary Table 1:** **(A)** Mature miRNA sequences of each family are shown. Bolded letters denote the seed sequence of each miRNA. **(B)** Sequences and product information of each inhibitor used in this study is shown. Bolded letters denote anti-miR sequence that is perfectly complimentary to the seed sequence of its corresponding miRNA family.


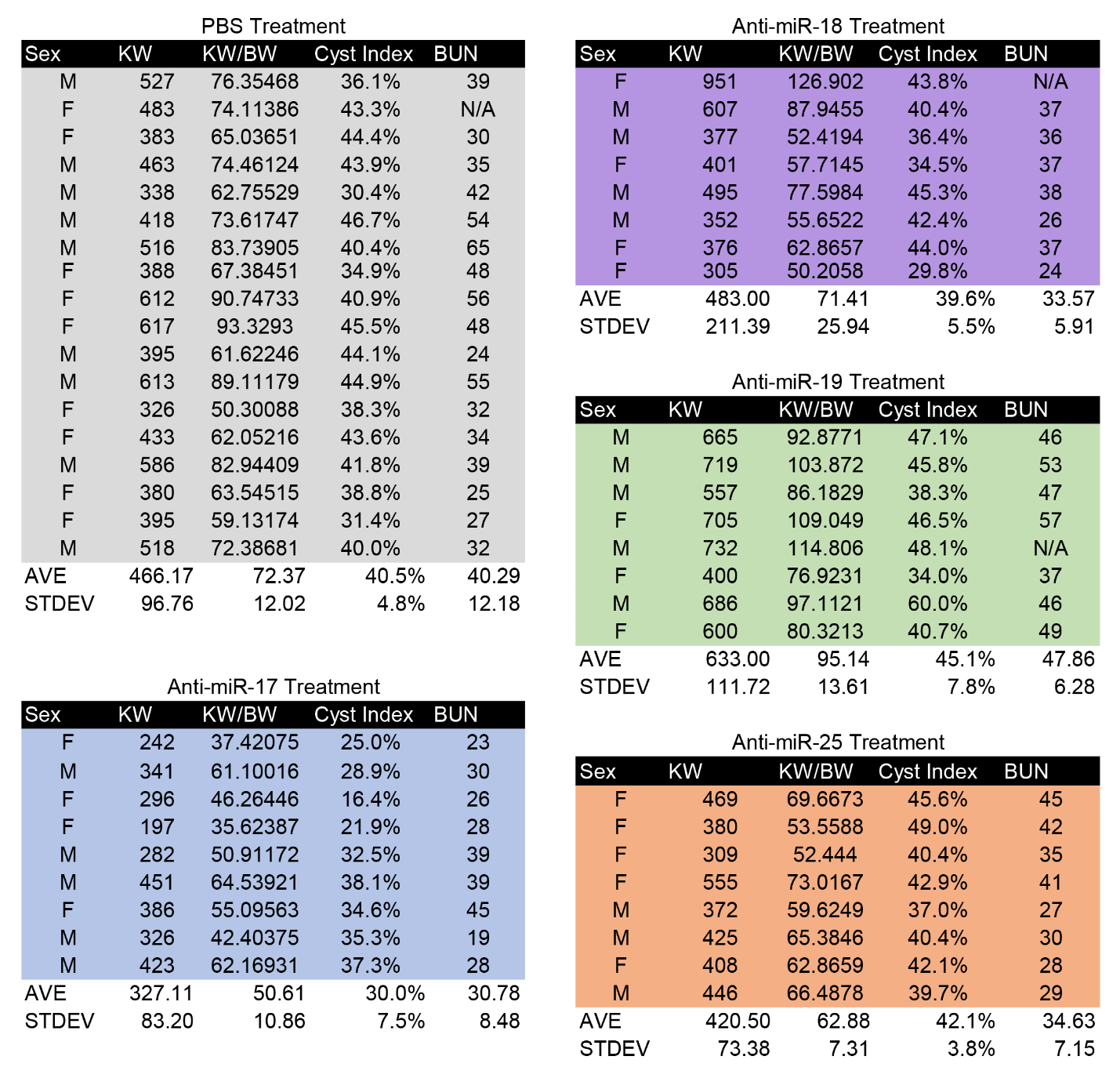


**Supplementary Table 2**: Full information of all mice used in this study

|  | | | | | | |
| --- | --- | --- | --- | --- | --- | --- |
| *Kim1* | F | 5'-AGCAGTCGGTACAACTTAAAGG-3' |  | *Atp5e* | F | 5'-CAGGCTGGACTCAGCTACATC-3' |
|  | R | 5'-AGAGTTCTCTATCGTCAAGGACA-3' |  |  | R | 5'-GTTCGCTTTGAACTCGGTCTT-3' |
| *Ngal* | F | 5'-GCAGGTGGTACGTTGTGGG-3' |  | *Acta2* | F | 5'-GTCCCAGACATCAGGGAGTAA-3' |
|  | R | 5'-CTCTTGTAGCTCATAGATGGTGC-3' |  |  | R | 5'-TCGGATACTTCAGCGTCAGGA-3' |
| *Ppara* | F | 5'-CCTCAAAGTCTGAGCGGTCT-3' |  | *Col1a1* | F | 5'-GCTCCTCTTAGGGGCCACT-3' |
|  | R | 5'-CTAACCTTGGGCCACACCT-3' |  |  | R | 5'-CCACGTCTCACCATTGGGG-3' |
| *Ppargc-1a* | F | 5'-GTGAACATTCAAAGCAGCAGAG-3' |  | *Tgfb2* | F | 5'-CTTCGACGTGACAGACGCT-3' |
|  | R | 5'-TTCTTCGTACAGCCATCAAAAA-3' |  |  | R | 5'-GCAGGGGCAGTGTAAACTTATT-3' |
| *Fbp1* | F | 5'-CACCGCGATCAAAGCCATCT-3' |  | *Ifng* | F | 5'-ATGAACGCTACACACTGCATC-3' |
|  | R | 5'-AGGTAGCGTAGGACGACTTCA-3' |  |  | R | 5'-CCATCCTTTTGCCAGTTCCTC-3' |
| *G6pc2* | F | 5'-CAGGAGGACTACCGGACTTAC-3' |  | *Ccl5* | F | 5'GCTGCTTTGCCTACCTCTCC-3' |
|  | R | 5'-TCAACTGAAACCAAAGTGGGA-3' |  |  | R | 5'-TCGAGTGACAAACACGACTGC-3' |
| *Ndufv1* | F | 5'-TTTCTCGGCGGGTTGGTTC-3' |  | *Ccl22* | F | 5'CCTTTGACTCCTTGCCTGTG-3' |
|  | R | 5'-GGTTGGTAAAGATCCGGTCTTC-3' |  |  | R | 5'-TCACTTTGTATGGCCCTTCC-3' |
| *Ndufa2* | F | 5'-TTGCGTGAGATTCGCGTTCA-3' |  | *Il6* | F | 5'-TAGTCCTTCCTACCCCAATTTCC-3' |
|  | R | 5'-ATTCGCGGATCAGAATGGGC-3' |  |  | R | 5'-TTGGTCCTTAGCCACTCCTTC-3' |
| *Etfa* | F | 5'-GCCTCATTGCTCCGTTTTCAG-3' |  | *Mip2* | F | 5’-GCTGGCCACCAACCACCAG-3’ |
|  | R | 5'-GCTACTAAGCAGGACACTTCAC-3' |  |  | R | 5’-AGCGAGGCACATCAGGTA-3’ |
| *Etfdh* | F | 5'-GTGCGACTAACCCTGTC-3' |  | *Arg1* | F | 5'-CTCCAAGCCAAAGTCCTTAGAG-3' |
|  | R | 5'-GGATGAACAGTGTAGTGAGTGG-3' |  |  | R | 5'-AGGAGCTGTCATTAGGGACATC-3' |
| *Cox5a* | F | 5'-GCCGCTGTCTGTTCCATTC-3' |  | *Mrc1* | F | 5'-CTCTGTTCAGCTATTGGACGC-3' |
|  | R | 5'-GCATCAATGTCTGGCTTGTTGAA-3' |  |  | R | 5'-CGGAATTTCTGGGATTCAGCTTC-3' |

**Supplementary Table 3:** Sequences of primers used in the current study

**Uncropped Western Blots**
